# Keratinocytes with DNA Aberration Induced by UVB Become Susceptible to Ferroptosis

**DOI:** 10.3390/antiox15080966

**Published:** 2026-08-03

**Authors:** Yuliya D. Smirnova, Philipp Sabler, Adelheid Weidinger, Johannes Grillari, Peter Dungel, Andrey V. Kozlov

**Affiliations:** 1 Ludwig Boltzmann Institute for Traumatology, The Research Center in Cooperation with AUVA, 1200 Vienna, Austria; dyd16@mail.ru (Y.D.S.); philipp.sabler@trauma.lbg.ac.at (P.S.); adelheid.weidinger@trauma.lbg.ac.at (A.W.); johannes.grillari@trauma.lbg.ac.at (J.G.); 2 Laboratory of Metagenomics and Food Biotechnology, Voronezh State University of Engineering Technologies, 394036 Voronezh, Russia; 3 Institute for Molecular Biotechnology, University of Natural Resources and Life Sciences, 1190 Vienna, Austria; 4 Austrian Cluster for Tissue Regeneration, 1200 Vienna, Austria

**Keywords:** keratinocytes, ferroptosis, UVB, cyclobutane pyrimidine dimers, RSL3, erastin

## Abstract

Keratinocytes are key epidermal cells that are highly susceptible to ultraviolet (UV)-induced damage, which can lead to DNA mutations and the development of malignancies. In parallel, UV radiation induces lipid peroxidation (LPO), potentially facilitating the activation of ferroptosis, a form of programmed cell death. We hypothesized that UV-mediated DNA damage, resulting in the formation of cyclobutane pyrimidine dimers (CPDs), occurs preferentially in cells with elevated LPO levels, and that mild induction of ferroptosis in proliferating keratinocytes selectively eliminates cells with high CPD levels. A human keratinocyte cell line was exposed to UVB radiation and subsequently treated with the ferroptosis inducers RSL3 and erastin. Cell death was assessed using LDH analysis, LPO was measured using the fluorescent probe BODIPY™ 581/591 C11, and CPD formation was quantified by ELISA. Using different doses of UVB, we confirmed UVB irradiation simultaneously increases the cell death rate and LPO and CPDs levels in proliferating keratinocytes. Mild induction of ferroptosis in these cells led to a slight increase in the cell death rate and simultaneously to a drastic reduction in CPD levels, suggesting that there is a specific pool of cells predominantly susceptible to UVB in terms of DNA damage and LPO induction. Our findings support our hypothesis that induction of ferroptosis in proliferating keratinocytes exposed to UVB radiation preferentially eliminates cells with elevated CPD levels and may therefore serve as a protective mechanism against UV-induced carcinogenesis.

## 1. Introduction

Keratinocytes are the most abundant cell type in the skin. They continuously proliferate and play a critical role in maintaining the skin barrier, producing keratin and facilitating wound healing. However, they are also implicated in pathological conditions such as psoriasis and keratosis and can undergo malignant transformation into carcinoma, particularly following exposure to ultraviolet (UV) irradiation.

Recently, keratinocytes have been associated with ferroptosis, another form of programmed cell death driven by iron-mediated oxidative stress [1]. The pathophysiological relevance of this mechanism remains controversial.

It has been suggested that ferroptosis contributes to the dysfunction of diabetic keratinocytes, and that targeting ferroptosis may represent a therapeutic strategy to improve diabetic wound healing [2,3]. Ferroptotic stress may also play a dual role, e.g., in psoriasis by promoting inflammation [1,4], while in certain contexts limiting keratinocyte hyperproliferation. The ferroptosis inducer RSL3 has been shown to ameliorate psoriatic symptoms by improving abnormal keratinocyte function [5]. As a glutathione peroxidase 4 (GPX4) inhibitor, RSL3 reduces keratinocyte proliferation, suggesting that ferroptosis induction may exert a protective effect in psoriasis vulgaris [5].

In addition, inhibition of ferroptosis has been shown to promote the growth of cutaneous squamous cell carcinoma [6], suggesting a potentially beneficial role for ferroptosis in skin cancer treatment.

UVB irradiation has also been demonstrated to induce ferroptosis in keratinocytes. Inhibition of ferroptosis reduces necroinflammation in UVB-irradiated mouse skin [4]. Moreover, UVB significantly decreases keratinocyte viability and increases apoptosis and reactive oxygen species (ROS) levels, whereas treatment with ferrostatin-1 (Fer-1) restores cell viability and reduces apoptosis and ROS production [7]. Similar to psoriasis, it remains unclear whether these effects are ultimately beneficial or detrimental in the context of UV-induced skin cancer.

We hypothesize that ferroptosis plays a dual role following UVB exposure by preventing the survival of damaged keratinocytes while simultaneously preventing survival of cells with DNA aberrations, which may contribute to cancer development. This assumption is based on the idea that DNA damage and susceptibility to exogenous ferroptosis inducers may occur in the same cells.

Exposure to UV radiation from sunlight or artificial sources damages DNA in skin cells, leading to mutations that may result in cancer. UVA (320–400 nm) penetrates deeply into the dermis and primarily causes damage through the generation of ROS, affecting DNA as well as other intracellular and extracellular targets. In contrast, UVB (290–320 nm) is primarily responsible for sunburn and directly damages DNA by inducing the formation of cyclobutane pyrimidine dimers (CPDs) and 6–4 photoproducts [8]. UVB is approximately 200 times more efficient than UVA in causing direct DNA damage [9]. Cellular susceptibility to UV irradiation is also strongly influenced by the phase of the cell cycle.

Induction of ferroptosis in keratinocytes has been established in numerous studies. It can be triggered by agents such as erastin and RSL3 [5,10,11], while ferrostatin-1 suppresses ferroptosis-related changes in erastin-treated keratinocytes [1,12,13].

Therefore, the aim of our study was to examine how the induction of ferroptosis in keratinocytes exposed to UVB irradiation affects cell death rates and the formation of CPDs.

## 2. Materials and Methods

### 2.1. Cell Culture Experimental Design

For our experiments, we used the human keratinocyte-derived cell line, NHEK/SVTERT3–5 (Evercyte GmbH, Vienna, Austria), which comprises human epidermal keratinocytes with epithelial morphology that were previously used as a model for primary keratinocytes. This cell line was immortalized using the ectopic expression of hTERT and SV40 large T antigen, while still keeping the typical functions of keratinocytes, as they are commonly used for skin equivalent models for drug response and wound healing studies [14,15,16,17]. Cells were grown in KGM™-Gold BulletKit™ medium (Lonza; Lot No.: Basal Medium: 00195130; SingleQuots™: 00192152). The 500 mL of medium was additionally supplemented with 50 µL of 1 M CaCl_2_ and 500 µL of G418 instead of gentamicin. The final Ca^2+^ concentration was 0.1 mM, which supports keratinocyte proliferation. NHEK_SVTERT3–5 line has a constant population doubling time of 48–60 h (https://evercyte.com/wp-content/uploads/2021/05/NHEK_SVTERT3_5_leaflet.pdf, accessed on 20 July 2026). The cells were passaged after reaching 80–100% confluence, usually twice per week, split either 1:2 or 1:3. After treatment with erastin or RSL3 (concentrations are indicated in the figure legends), the cells were incubated for 24 h and then exposed to UVB irradiation. For irradiation, the cell culture plate was placed on a heat block, and the temperature of the culture medium was monitored using an infrared thermometer and maintained at 37 °C throughout the procedure. In a preliminary calibration experiment, six positions (corresponding to one half of a 12-well plate) were selected for UVB exposure (they were exposed to equal irradiation dose); the remaining wells were not used. Before each experiment, the UVB lamp was calibrated, and the exposure time required to deliver the desired UVB dose was calculated. An exposure time of approximately 60 s was required to achieve a dose of 10 kJ/m^2^.

The experimental workflow was as follows. Cells were seeded into 12-well plates and cultured for 48 h. On day 3, the cells were treated with the indicated ferroptosis inducers and incubated for an additional 24 h. Before irradiation, the culture medium was carefully removed and retained, and the cells were rinsed with PBS. Cells assigned to the irradiation group were exposed to UVB while covered with PBS. Immediately after irradiation, the PBS was removed, and the original conditioned medium was returned to the corresponding wells. Control cells underwent the same procedure, except that they were incubated with PBS for 2–3 min without UVB exposure before the original medium was restored. Both the irradiated and control plates were then incubated for an additional 16 h, after which the samples were harvested for subsequent analyses. The data generated from each weekly run were therefore considered to represent an independent experiment.

### 2.2. Sample Collection

After the final incubation of an experiment, the medium containing released lactate dehydrogenase (LDH) and the live adherent cells were separated and stored for further LDH measurement and CPD analysis. The medium was transferred to microcentrifuge tubes and frozen with liquid nitrogen. The plates were put into an ultrasonic water bath and sonicated at the lowest setting for 60 s at room temperature to loosen the cells. The plate’s wells were scraped with the rubber tip of a syringe piston; the resulting cell suspension was transferred into microcentrifuge tubes and frozen with liquid nitrogen. Then, they were kept for max. 2 weeks at −20 °C. One portion of the frozen cells was used to determine the LDH levels in the medium and cell lysate. The second portion of the samples was used to extract DNA from the entire population of cells (both floating in medium and adherent (pellet) cells) by pooling both frozen samples. In these samples, we determined the quality of DNA using electrophoresis and analyzed CPDs in the extracted DNA. The third analytical setup used live cells, which were treated with BODIPY, a fluorescence reporter detecting oxidized and non-oxidized lipids separately, and examined by laser scanning microscopy. The ratio of oxidized/non-oxidized lipids was used as the measure of lipid peroxidation.

### 2.3. Erastin/RSL3-Treatments

The cells were treated with either erastin (21.33, 10.67, 5.33 and 2.67 μM) or RSL3 (5.33, 2.67, 1.33 and 0.67 μM) and incubated for 24 h at 37 °C followed by UV irradiation, or for 40 h and then harvested. The sources of reagents were RSL3 (S8155–5 mg, Eubio, Vienna, Austria) and erastin (S7242–5 mg, Eubio, Austria).

### 2.4. Irradiation

As the UV source, we used a Dermfix 2000SX device equipped with a TL/01-type 311 nm narrowband UVB lamp. It was calibrated with a Waldmann UV meter, using a UV21 calibration sensor. Although their emission spectrum of TL/01 is similar to that of Waldmann UV21 lamps, TL/01 lamps have a narrower bandwidth. Because of this, the irradiance measurements were corrected for the TL/01 spectrum using the relative irradiance reported by Taylor et al. [18], showing that the effective irradiance of the TL/01 lamps is approximately 30% higher than the UV21. Multiple components of cell culture medium, such as amino acids absorb the UVB light around 310 nm, with reported absorbance values of approximately 1 absorbance units (AU) [19]. According to the Beer–Lambert law, an absorbance of 1.0 AU corresponds to approximately 10% transmission. To avoid such a high loss of irradiation, we changed the medium to PBS for the time of irradiation.

### 2.5. LDH-Measurement

We selected the LDH assay to evaluate the amount of dead and dying cells, because a substantial body of evidence supports the validity of LDH release as a marker of cell death of keratinocytes. Several studies have demonstrated a positive correlation between LDH release and other cell death assays, such trypan blue exclusion [20,21,22]. In addition, an inverse correlation between LDH release and cell viability assessed by the MTT assay has been reported in keratinocytes [23,24]. Similar to trypan blue staining, the MTT assay evaluates only adherent metabolically active cells and does not account for detached cells.

Furthermore, the LDH assay has been extensively and successfully employed to quantify UV-induced cell death in keratinocytes [22,25,26,27,28,29,30,31], further supporting its suitability for the present study.

In both the medium and cell homogenates, we determined the activity of LDH. The ratio of LDH determined in the medium to the total amount of LDH in the sample (medium plus cell homogenate) was considered as the relative portion of dead and dying cells. LDH activity measurements were performed as previously described by Weidinger et al. [32] with modifications described by Smirnova et al. [33]. Briefly, a 25% sample volume of RIPA buffer was added to the cell suspension samples, mixed by pipetting up and down, and incubated on ice for 20 min. Then, 150 µL of RT-PBS was added. The samples were then pipetted into a 96-well plate. LDH release was measured by mixing 30 µL of cell culture supernatant (or cell homogenate) with 100 µL of LDH assay reagent containing 110 mM lactic acid, 1350 mM nicotinamide adenine dinucleotide (NAD), 290 mM N-methylphenazonium methyl sulfate (PMS), 685 mM 2-(4-iodophenyl)-3-(4-nitrophenyl)-5-phenyl-2H-tetrazolium chloride (INT), and 200 mM Tris (pH 8.2). Absorbance changes were read kinetically at 492 nm for 30 min using a Polarstar Omega plate reader, software version 5.11 (BMG Labtech, Ortenberg, Germany). LDH activity values were determined as the maximum rate of NADH formation, determined by the changes of absorbance at 492 nm (A492/dt (min)). Measurements were carried out in triplicate. Data were exported to Microsoft Excel (Microsoft Corp., Redmond, WA, USA) for processing and analyzed in Prism 9.2 (Graphpad, San Diego, CA, USA).

### 2.6. L-ROS-Staining

For lipid peroxidation (LPO) analysis, BODIPY™ 581/591 C11 (Invitrogen, Waltham, MA, USA), a highly sensitive fluorescent probe, was used. The probe easily integrates into cell membranes, and lipid oxidation causes a fluorescence shift: the unoxidized form fluoresces in the red spectrum, while the oxidized form fluoresces in the green. This allows for easy monitoring of the process in real time. Here, 500 µL of medium from each well of confluent plates was pipetted into Eppendorf tubes. Then, 2.5 microliters of BODIPY stock solution were added to each tube to achieve a BODIPY concentration of 5 μM. After thorough mixing with a vortex, the medium was returned to the appropriate wells. The plates were incubated for 30 min at 37 °C and 5% CO_2_. The medium was removed, and the cells were washed with 1 mL of PBS. Then, 500 µL of HBSS was added to each well. LPO was then immediately assessed using a Zeiss LSM510 Meta laser scanning confocal microscope (Zeiss, Oberkochen, Germany) at 10× magnification. An excitation/emission wavelength of 581/591 nm was used to detect the unoxidized state, and an excitation/emission wavelength of 488/510 nm was used for the oxidized state. Three images per well were acquired using ZEN 2009 (version 6.0.303, Zeiss, Germany) and then analyzed using ImageJ (version 1.53, US National Institutes of Health, Bethesda, MD, USA).

### 2.7. DNA Isolation

DNA was isolated from the cells using an Invitrogen PureLinkTM Genomic DNA Mini Kit (Thermo Fisher, Waltham, MA, USA; Lot-No.: 2535424), as recommended by the manufacturer. The amount of total DNA obtained from the samples was determined using a Nanodrop One/OneC. Quality was assessed by agarose gel electrophoresis.

### 2.8. CPD-Analysis

CPDs were measured in duplicate and in a random order using an OxiSelect Cellular UV-Induced DNA Damage ELISA Kit (CPD) (Cell Biolabs, San Diego, CA, USA, Lot-No.: 3221514), according to the manufacturer’s instructions. Briefly, equal amounts of DNA (4 µg/mL) were added into each ELISA well according to the manufacturer’s protocol, and the DNA was denatured and then immobilized on the plate. The wells were treated with an CPD-specific antibody and incubated. Several washing cycles were performed. The HRP conjugate was added and incubated; then, the wells were thoroughly washed. Then, the substrate was added, and the absorbance was measured at 450 nm. The OD450 within each plate was normalized to the average values of cells treated with only 5 kJ/m^2^, and this value was taken as 100%.

### 2.9. Statistical Analysis

The data are shown as the mean + SEM, outliers were excluded with ROUT [Q = 5%], and statistical significance was analyzed by One-Way ANOVA followed by Holm–Šídák’s multiple comparisons test.

## 3. Results

**Figure 1:**
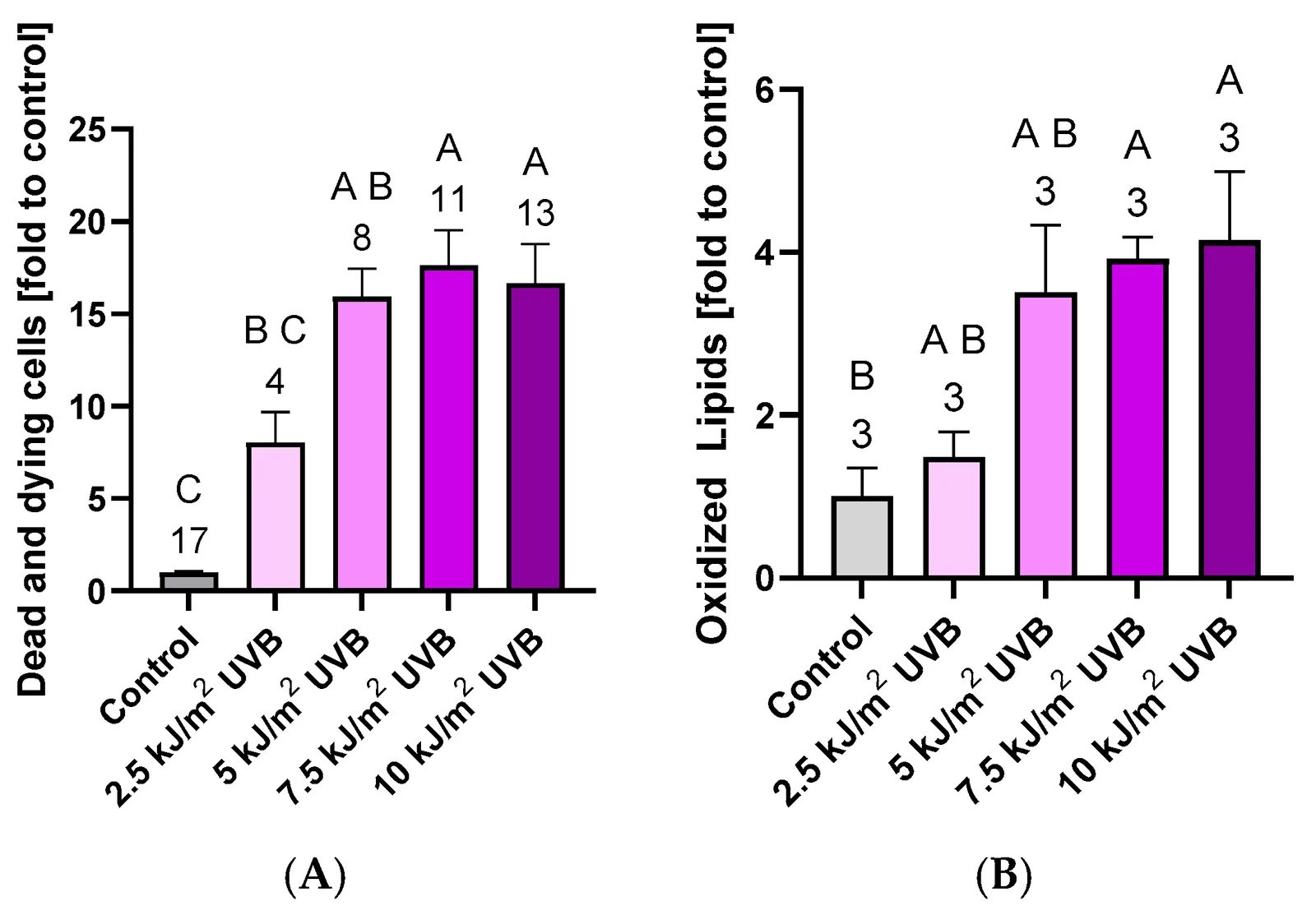
(**A**) Effect of different doses of irradiation on the rate of cell death. Cell death was quantified by measuring the proportion of lactate dehydrogenase (LDH) released into the incubation medium relative to the total LDH content in each well (medium + cell pellet). The baseline cell death rate in the control group was 3.16 ± 0.15% (mean ± SEM). All experimental values are presented as fold changes relative to this control. (**B**) Effect of UVB on the activation of lipid peroxidation (LPO). LPO was determined by fluorescent dye BODIPY using images obtained by confocal microscopy. Data are presented as the mean ± SEM, and the number of independent biological replicates is indicated above the corresponding bars. Statistical analyses were performed using one-way ANOVA followed by Holm–Šídák’s multiple comparisons test. Statistical differences are indicated by Compact Letter Display (CLD) labeling at *p* < 0.05.

After establishing our experimental model, we determined the range of UV doses that induce cell death and lipid peroxidation in keratinocyte cultures. Increasing levels of UVB irradiation resulted in a parallel rise in both cell death and lipid peroxidation, indicating a dose-dependent cytotoxic and oxidative response of keratinocytes to UVB (Figure 1). Considering that DNA damage may not directly correlate with the extent of cell death and lipid peroxidation, we selected UVB doses of 5 kJ/m^2^ and 10 kJ/m^2^ for subsequent experiments. In subsequent experiments, we aimed to identify a reliable pharmacological inducer of ferroptosis in keratinocyte cultures.

**Figure 2:**
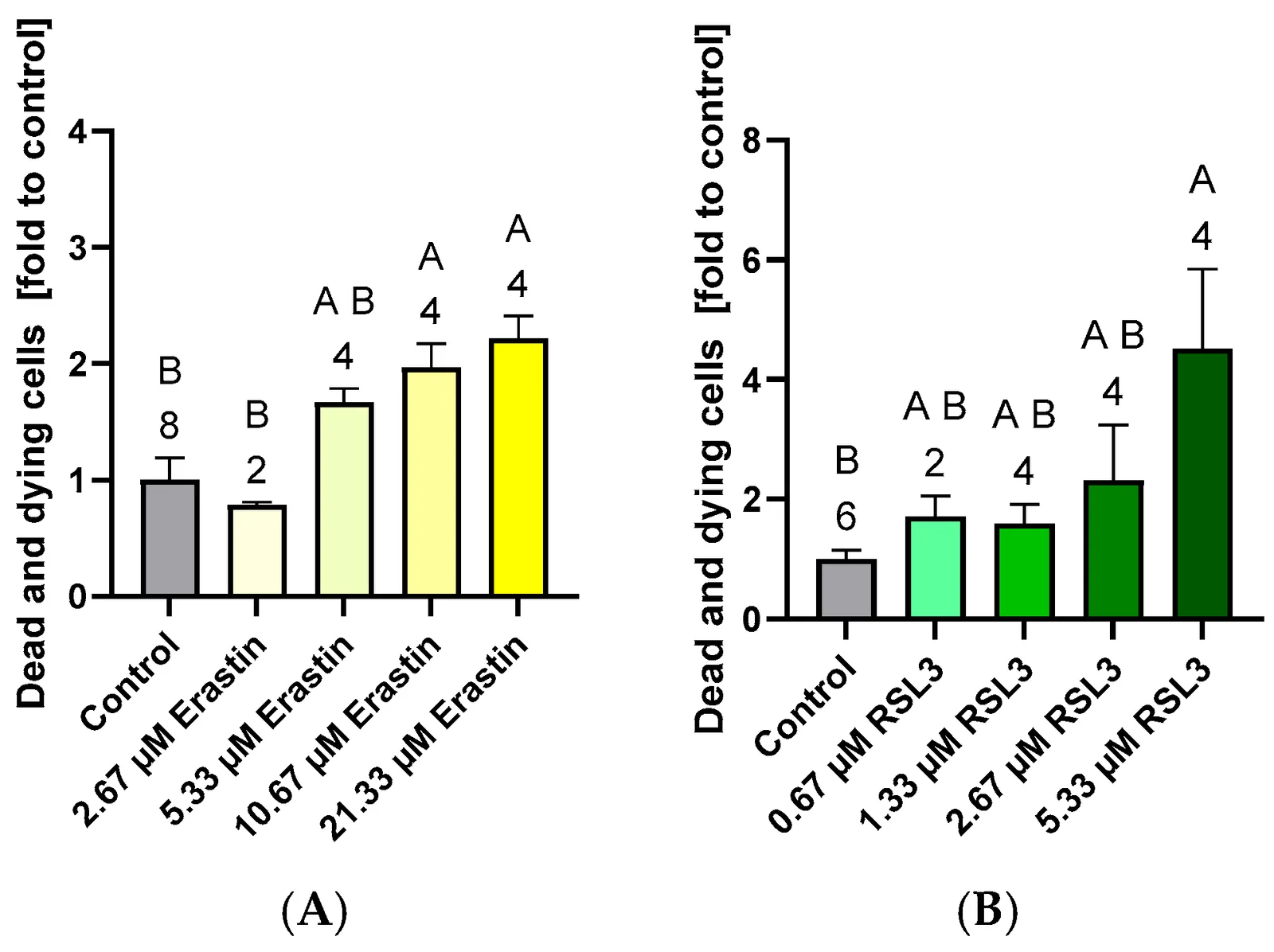
Effect of erastin, an inhibitor of cystine–glutamate antiporter and RSL3, an inhibitor of GPX4, on the rate of cell death. (**A**). Effect of erastin on the cell death rate. (**B**). Effect of RSL3 on the cell death rate. Cell death was quantified by measuring the proportion of lactate dehydrogenase (LDH) released into the incubation medium relative to the total LDH content in each well (medium + cell pellet). The baseline cell death rate in the control group was 8.61 ± 3.60 (mean ± SEM). All experimental values are presented as fold changes relative to this control value. Data are presented as the mean ± SEM, and the number of independent biological replicates is indicated above the corresponding bars. Statistical analyses were performed using one-way ANOVA followed by Holm–Šídák’s multiple comparisons test. Statistical differences are indicated by Compact Letter Display (CLD) labeling at *p* < 0.05.

We tested two well-established ferroptosis inducers in keratinocyte cultures, RSL3 and erastin. Increasing concentrations of each compound were applied to the cultures until a consistent, yet moderate, level of cell death was achieved, preferably at lower inducer concentrations. Both erastin and RSL3 induced cell death; however, RSL3 proved to be more effective, eliciting higher levels of cell death at lower concentrations (Figure 2). Based on these results, RSL3 was selected for subsequent experiments. We then investigated the combined effects of UVB irradiation and RSL3 on cell death and LPO. First, we examined LPO.

**Figure 3:**
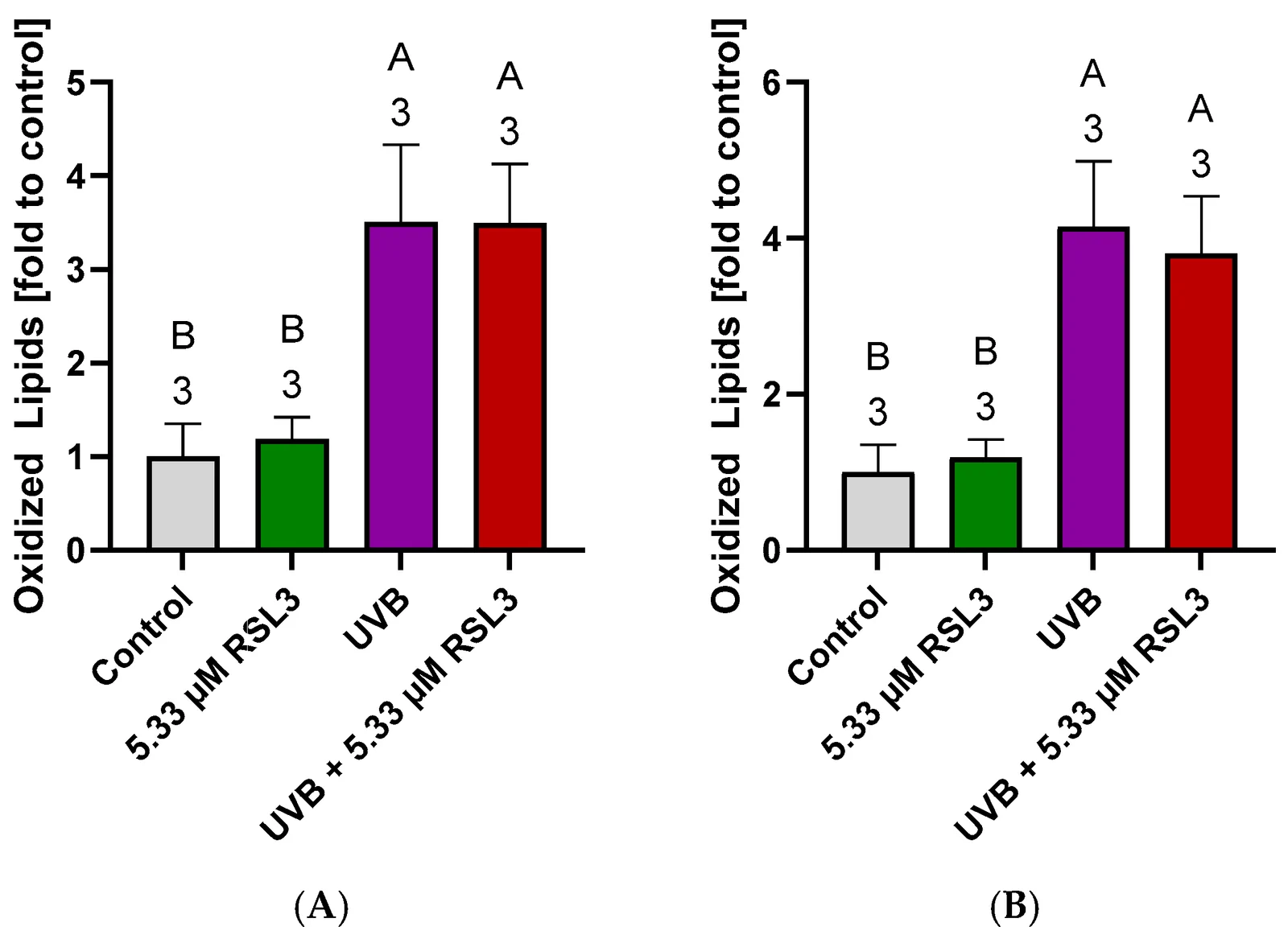
Combined effect of UVB irradiation and inducers of ferroptosis on the levels of oxidized lipids. (**A**). Effect of low (5 kJ/m^2^) intensities of UVB irradiation. (**B**). Effect of high (10 kJ/m^2^) intensities of UVB irradiation. Data are presented as the mean ± SEM, and the number of independent biological replicates is indicated above the corresponding bars. Statistical analyses were performed using one-way ANOVA followed by Holm–Šídák’s multiple comparisons test. Statistical differences are indicated by Compact Letter Display (CLD) labeling at *p* < 0.05.

In these experiments, we used UVB doses of 5 and 10 kJ/m^2^ to induce and examined the effects of RSL3 (Figure 3). The LPO levels were slightly higher at 10 kJ/m^2^ compared to 5 kJ/m^2^; however, this difference was not statistically significant, suggesting that RSL3 treatment did not further increase the LPO levels. One possible explanation is that cells experiencing additional RSL3-induced LPO undergo ferroptotic cell death and detach from the culture, thereby no longer contributing to the measured LPO in the remaining adherent cells. To test this hypothesis, we next evaluated the effect of RSL3 on UVB-induced cell death.

**Figure 4:**
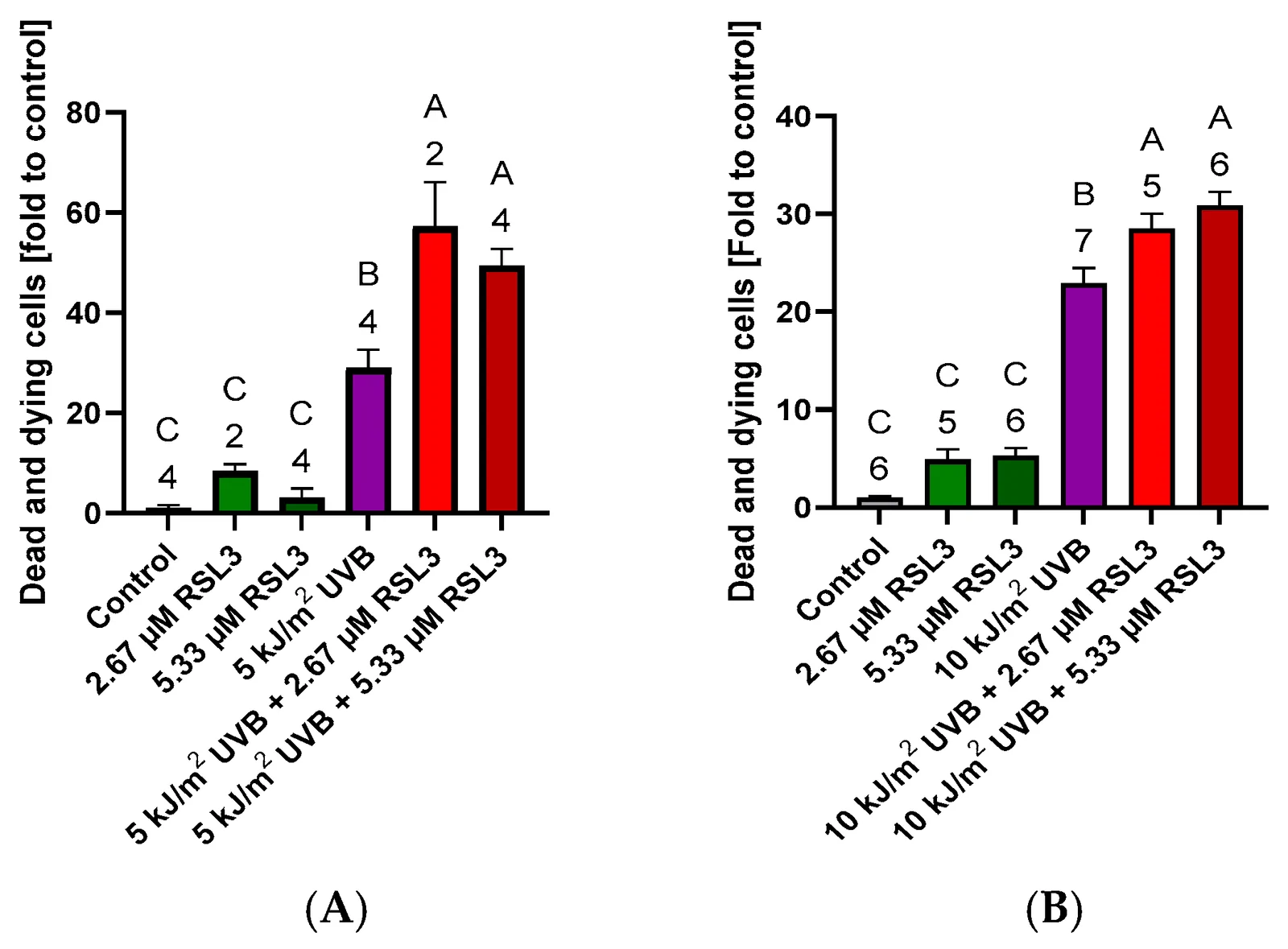
Combined effect of UVB irradiation and RSL3 on the rate of cell death. (**A**). Results obtained at 5 kJ/m^2^ UVB. (**B**). Results obtained at 10 kJ/m^2^ UVB irradiation. Cell death was quantified by measuring the proportion of lactate dehydrogenase (LDH) released into the incubation medium relative to the total LDH content in each well (medium + cell pellet). The baseline cell death rates in the control groups were 1.15 ± 0.67 and 2.55 ± 0.48 (mean ± SEM) for A and B, respectively. Data are presented as the mean ± SEM, and the number of independent biological replicates is indicated above the corresponding bars. Statistical analyses were performed using one-way ANOVA followed by Holm–Šídák’s multiple comparisons test. Statistical differences are indicated by Compact Letter Display (CLD) labeling at *p* < 0.05.

As observed previously, although RSL3 did not affect the LPO levels, it significantly increased the rate of cell death. This increase was approximately two-fold at 5 kJ/m^2^ and 1.5-fold at 10 kJ/m^2^ (Figure 4). We did not find significant differences between induction of RSL-mediated cell death upon 5 kJ/m^2^ and 10 kJ/m^2^ of UVB irradiation. We next considered two options: RSL3-induced cell death occurred either randomly across the cell population or preferentially affected cells with DNA damage. If cell death were random, one would expect a proportional reduction in DNA aberrations—approximately twofold at 5 kJ/m^2^ and 1.5-fold at 10 kJ/m^2^ following RSL3 treatment. In contrast, if ferroptosis were preferentially induced in cells with DNA damage, a greater reduction in DNA aberrations than in overall cell death would be anticipated. To distinguish between these possibilities, we measured the levels of CPDs in our samples.

**Figure 5:**
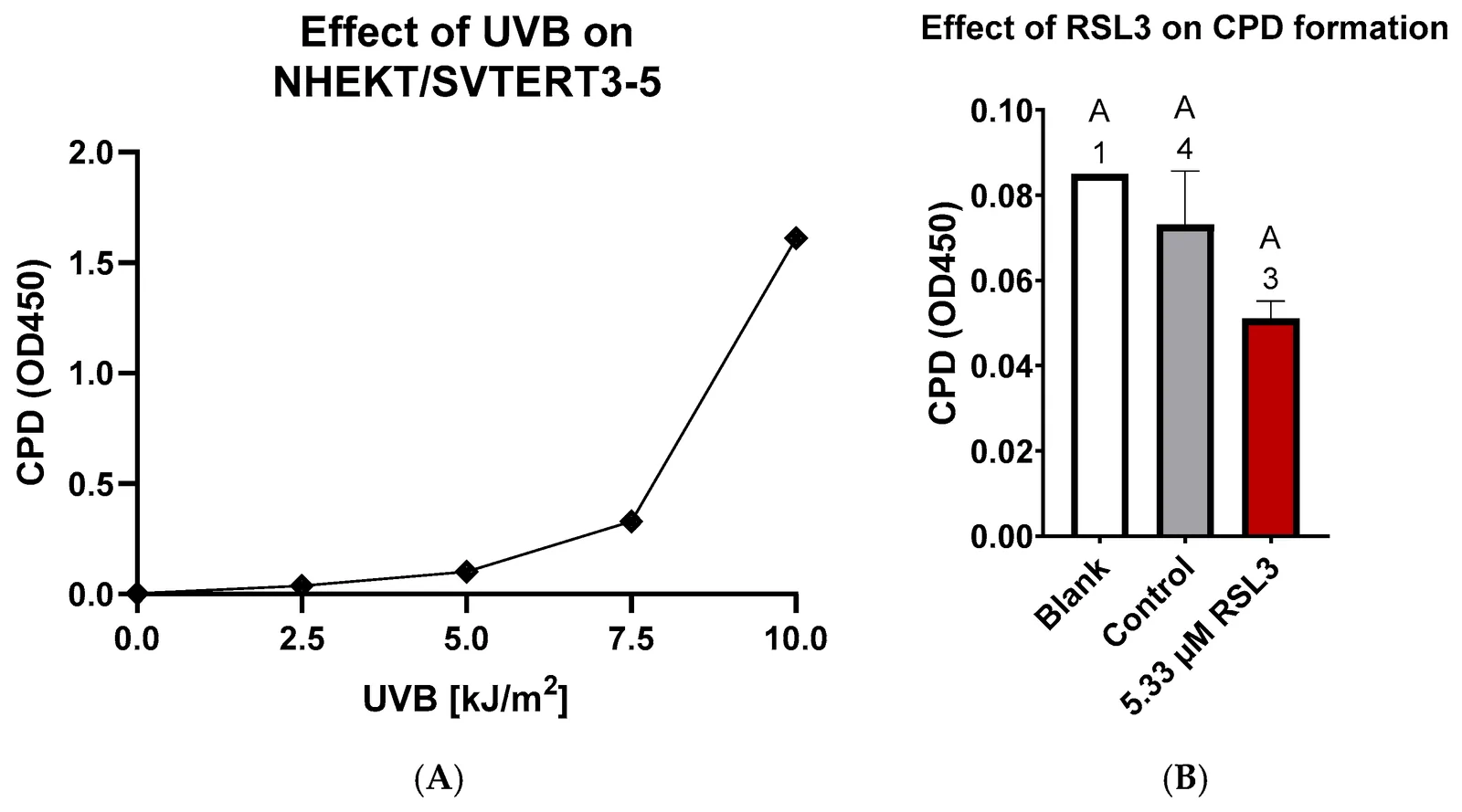

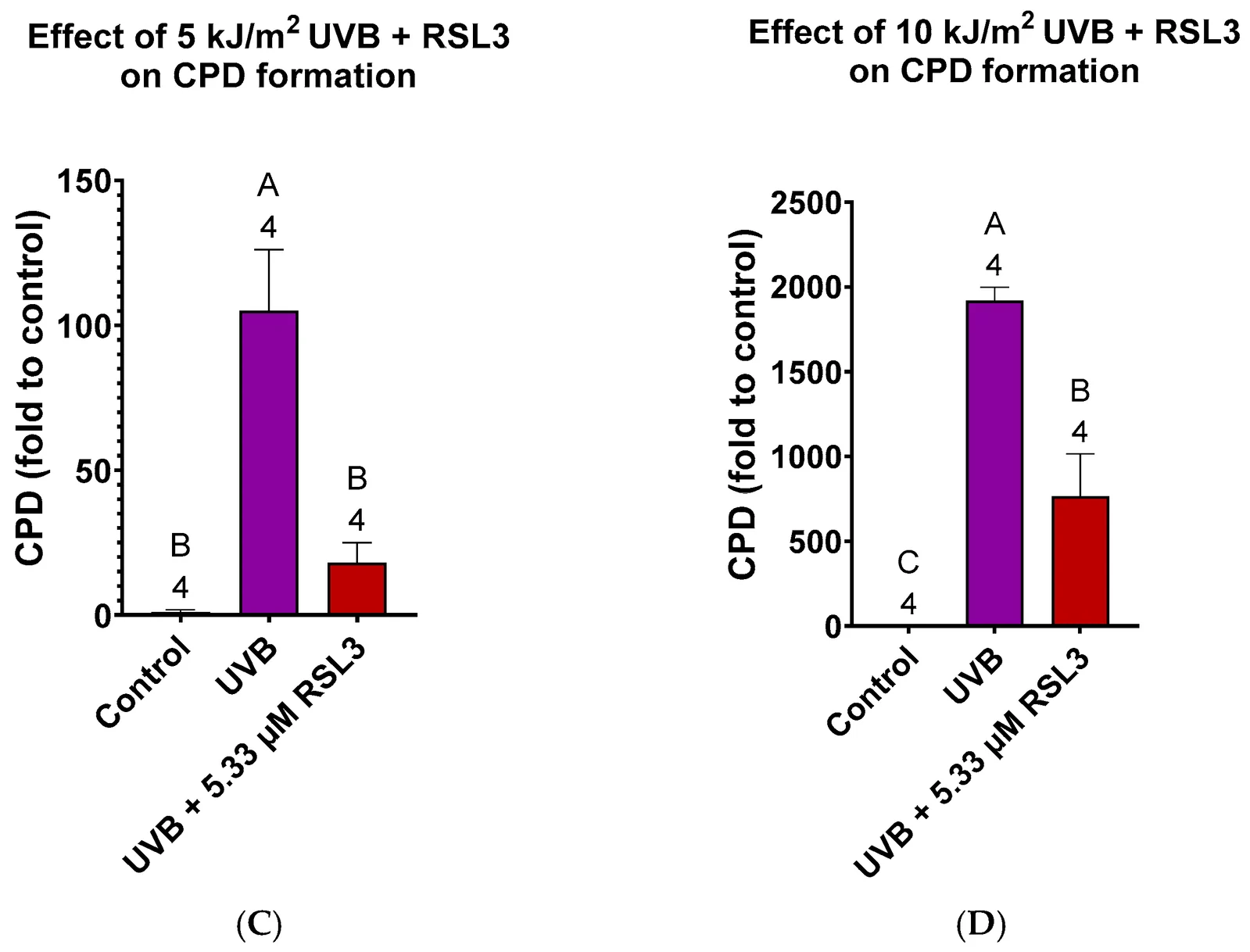
Effect of RSL3 on the formation of CPD in control cells and in cells subjected to UVB. (**A**). Effect of UVB on the formation of cyclobutane pyrimidine dimers (CPD). (**B**). RSL3 slightly but not significantly decreased the levels of CPD. (**C**). Effect of RSL3 on the accumulation of CPD in keratinocytes subjected to 5 kJ/m^2^ UVB. (**D**). Effect of RSL3 on the accumulation of CPD in keratinocytes subjected to 10 kJ/m^2^ UVB. Data are presented as the mean ± SEM, and the number of independent biological replicates is indicated above the corresponding bars. Statistical analyses were performed using one-way ANOVA followed by Holm–Šídák’s multiple comparisons test. Statistical differences are indicated by Compact Letter Display (CLD) labeling at *p* < 0.05.

First, we analyzed control samples and observed no significant effect of RSL3, although a trend toward reduced CPD levels was noted in its presence (Figure 5). In contrast, a markedly different pattern emerged following UVB irradiation. CPD levels increased dramatically—approximately 100-fold at 5 kJ/m^2^ and 2000-fold at 10 kJ/m^2^. RSL3 treatment substantially reduced the CPD levels under both conditions, with an approximately five-fold decrease at 5 kJ/m^2^ (17.3%) and a nearly three-fold decrease at 10 kJ/m^2^ (39.9%). These findings support our hypothesis that, following UVB irradiation, the induction of ferroptosis preferentially eliminates keratinocytes with DNA damage.

To further support our hypothesis, we repeated the key experiments examining the effects of ferroptosis induction on cell death and CPD levels using erastin. The results are presented in the Supplementary Materials. Supplementary Figures S1 and S2 demonstrate similar trends. Erastin reduced the CPD levels to a larger extent than would be expected based on the observed increase in cell death, although its overall effect was weaker compared to RSL3.

## 4. Discussion

The main finding of this study is that the formation of DNA aberrations in proliferating keratinocytes following UVB exposure coincides with their sensitivity to ferroptosis induction. We demonstrate that cells undergoing death contain a higher proportion of cyclobutane pyrimidine dimers (CPDs) compared to the average CPD level in the overall keratinocyte population. Our data suggest that cells susceptible to ferroptotic death harbor higher levels of UVB-induced CPDs than the average keratinocyte. Consequently, the selective elimination of these cells through mild induction of ferroptosis substantially reduces the overall CPD burden in the remaining keratinocyte population.

It is widely accepted that UVB radiation reaches the basal layer of the intact epidermis at a depth of approximately 100 µm, although its intensity may decrease to only a few percent of the incident power [34]. However, computer simulations based on Monte Carlo method and a skin tissue model suggest that UVB radiation can penetrate much deeper into the epidermis, reaching depths of up to 400 µm [35]. In contrast, UVA radiation penetrates the skin substantially deeper than UVB but is considerably less efficient at inducing CPDs. Specifically, exposure to the same radiant dose (1 J/m^2^) at 310 nm (UVB) produces approximately 100-fold more CPDs than exposure at 365 nm (UVA) [36]. The irradiation doses used in this study (5 or 10 kJ/m^2^) are fairly comparable with natural irradiation. It has been reported that, on a clear summer day in a north European country, approximately 1 kJ/m^2^ UVB can be received during 15–30 min of solar noon exposure, while substantially higher doses may occur in tropical regions [37].

We evaluated two ferroptosis inducers, RSL3 and erastin, and found that RSL3 is more effective in selectively eliminating CPD-containing cells in our model. This stronger effect may be attributed to the specific activation of ferroptosis by RSL3 through inhibition of GPX4, whereas erastin inhibits glutathione peroxidases more broadly and may also trigger additional redox-related pathways. Overall, our findings suggest a mechanistic link between cellular susceptibility to UVB-induced DNA damage and ferroptosis sensitivity.

We propose that this observed coincidence may be driven by cell cycle dynamics. Keratinocytes are rapidly proliferating and continuously progressing through the cell cycle. UVB irradiation is known to induce a G2/M arrest [38,39], and cells in this phase remain susceptible to UVB-induced damage for an extended period. Prolonged mitotic arrest can lead to the accumulation of damaged proteins and CPDs. In addition, structural rearrangements of chromosomes during the G2/M transition may further contribute to this effect.

Both CPD formation and ferroptosis are facilitated by excessive ROS generation and oxidative stress. It has been demonstrated that UVB exposure in human keratinocytes activates LPO, a process driven both by increased ROS production and by inhibition of antioxidant defense systems [40,41]. Furthermore, intracellular ROS levels are known to peak during the G2 and M phases of the cell cycle [42]. Together, these observations suggest that cells in G2 and mitosis are particularly susceptible to UVB-mediated oxidative stress. Consequently, cells carrying more UVB-induced DNA damage are also likely to exhibit elevated levels of lipid peroxides, rendering them more vulnerable to ferroptosis. This may explain our findings that the mild induction of ferroptosis preferentially affects cells with DNA aberrations.

Keratinocytes have several protection mechanisms preventing the formation of CPDs. One mechanism is the protection against UV by melanin. This pigment forms a protective “cap” over the nucleus, helping shield DNA from UV damage. It is synthetized in melanocytes. Keratinocytes take up melanin synthetized in melanocytes to create a shield over the own nucleus. The second mechanism is DNA repair. There are three major types of direct DNA repair: (a) reversal of UV-induced photolesions carried by photolyases, (b) repair of O-alkylated DNA by O6-alkylguanine-DNA alkyltransferases, and (c) repair of N-alkylated bases by AlkB family dioxygenase enzymes [43]. If repair is impossible, the cells may undergo apoptosis mediated by p53 activation [44] or by membrane death receptors or Bax translocation to mitochondria [45]. However, a population of keratinocytes with DNA damage may escape from all these mechanisms. Our experimental results suggest that the mild induction of ferroptosis can be an additional tool in preventing malignization. Thus, the mild induction of ferroptosis may be considered as an additional anticancer treatment upon UVB-mediated skin burn.

Our study highlights the fundamental feasibility of this approach. Further investigations are required to translate these mechanistic insights into a therapeutic strategy. Accordingly, this work does not aim to propose a novel therapeutic intervention, but rather to demonstrate the coincidence between cellular sensitivity to DNA damage and susceptibility to ferroptosis.

## 5. Limitations and Future Directions

This study focused on the interplay between induction of ferroptosis and DNA damage, whereas UVB irradiation also triggers numerous other cellular responses, including inflammatory signaling, stress responses, and changes in gene expression. The interaction between these processes and the mechanisms investigated here was not addressed in this study and warrants further studies.

A limitation of this study is that all experiments were performed using basal proliferating cells. Therefore, the findings cannot be directly extrapolated to untransformed or differentiated cells. Investigating whether these results also apply to those cell types will require further studies.

Further studies are required to determine whether RSL3 selectively eliminates cells carrying UVB-induced DNA aberrations through ferroptosis or other LPO-dependent cell death pathways. Future experiments should include Ferrostatin-1 or other selective ferroptosis inhibitors to establish whether inhibition of ferroptosis reverses the protective effects of erastin and RSL3 on UVB-induced DNA aberrations in both in vitro and in vivo models.

## 6. Conclusions

Our findings support our hypothesis that the induction of ferroptosis in proliferating keratinocytes exposed to UVB radiation preferentially eliminates cells with elevated CPD levels and may therefore serve as a protective mechanism against UV-induced carcinogenesis.

## Data Availability

All data files will be available from the digital data repository for published research of the Ludwig Boltzmann Society (https://creed.lbg.ac.at).

## Supplementary Materials

The following supporting information can be downloaded at https://www.mdpi.com/article/10.3390/antiox15080966/s1, This section contains information about experimental design in detail and additional experimental data obtained with erastin, another inducer of ferroptosis. It includes 4 figures. Figure S1. (A) The Dermfix UVB lamp (Dermfix GmbH, Westendorf, Austria) mounted in its holder, with the heat block positioned below and the plate placement marked with tape. (B) UV irradiation distribution measured using the Waldmann UV meter (Waldmann Lighting—Engineers of Light, Villingen-Schwenningen, Deutschland) for the UV21 settings. (C) Temperature (°C) plotted over time (minutes), showing the progression of the heat block alone in red and the metal plate supporting the 12-well plates in orange (n = 2). (D) UVB levels (kJ/m^2^) over time, with different colors representing the UV21 measurements at six designated spots, which were used for the treatment of the cells. Figure S2. After initial 4 day incubation, the cells had their medium changed, and/or were treated with Erastin/RSL3. After another 24 h of incubation, cells were irradiated with UVA or UVB, or kept in culture. After another 16 h of incubation, the cells were separated either frozen and separated into dead and live cells for LDH—measurements and DNA—ELISA, or stained in culture for radical microscopy. Figure S3. Combined effect of UVB irradiation and erastin on the rate of cell death. (A) Effect of erastin at low (5 kJ/m^2^) intensities of UVB irradiation. (B) Effect of erastin at high (10 kJ/m^2^) intensities of UVB irradiation. Cell death rate was determined by the release of LDH as described in method’s section. Figure S4. Effect of erastin on the elevated levels of CPD in keratinocytes exposed to 5 kJ/m^2^ (A) and 10 kJ/m^2^ (B) UVB irradiation. Inducers of ferroptosis decrease the levels of CPD by more than 2 fold, while the increase in the cell death rate upon treatment with erastin was not significant.
