# Supplementary material for: Keratinocytes with DNA Aberration Induced by UVB Become Susceptible to Ferroptosis
Source: Antioxidants (Basel). 2026 Aug 3;15(8):966. doi: 10.3390/antiox15080966 (PMC13509215; doi:10.3390/antiox15080966)
Supplement: Supplementary file 1 [file antioxidants-15-00966-s001.zip › antioxidants-4354632-supplementary.pdf]

**Supplementary Materials:**

**Figure S1** shows experimental design in detail. Preliminary experiments ensured that we do not have bias effects of temperature and differences in the irradiation doses between the experimental chambers. **Figure S2** shows general experimental design, used for analysis of the samples.

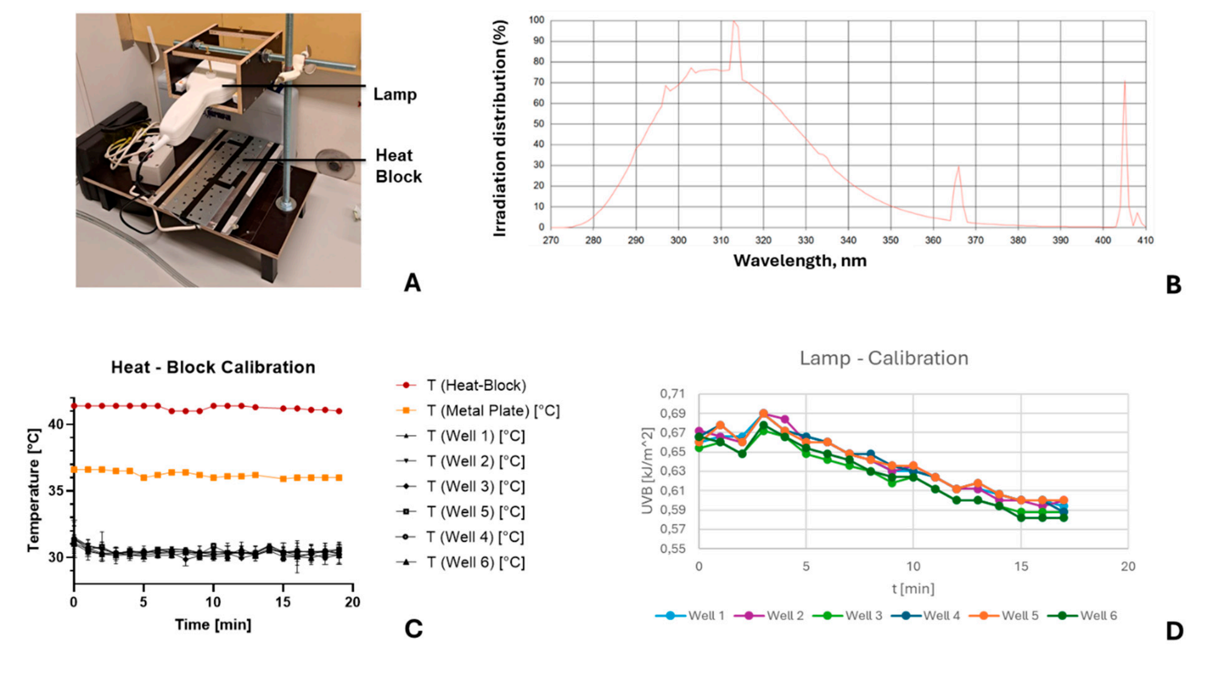


**Figure S1. (A)** The Dermfix UVB lamp (Dermfix GmbH, Westendorf, Austria) mounted in its holder, with the heat block positioned below and the plate placement marked with tape. **(B)** UV irradiation distribution measured using the Waldmann UV meter (Waldmann Lighting – Engineers of Light, Villingen-Schwenningen, Deutschland) for the UV21 settings. **(C)** Temperature (°C) plotted over time (minutes), showing the progression of the heat block alone in red and the metal plate supporting the 12-well plates in orange (n = 2). **(D)** UVB levels (kJ/m²) over time, with different colors representing the UV21 measurements at six designated spots, which were used for the treatment of the cells.

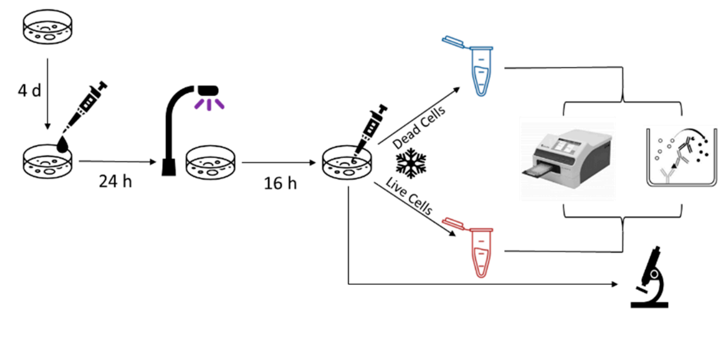


**Figure S2.** After initial 4 day incubation, the cells had their medium changed, and/or were treated with Erastin/RSL3. After another 24 hours of incubation, cells were irradiated with UVA or UVB, or kept in culture. After another 16 hours of incubation, the cells were separated either frozen and separated into dead and live cells for LDH – measurements and DNA – ELISA, or stained in culture for radical microscopy.

The main part of experiments was done with the specific ferroptose inducer RSL3. The critical experiments we have repeated with unspecific GPX inhibitor erastin. The results were very similar with those obtained with RSL3, but less pronounced. Erastin increased the mean values of cell death rate, but this increase was not significant (**Figure S3**). Simultaneously, the decrease in CPD levels was significant only upon irradiation with 10 kJ/m² (**Figure S4**).

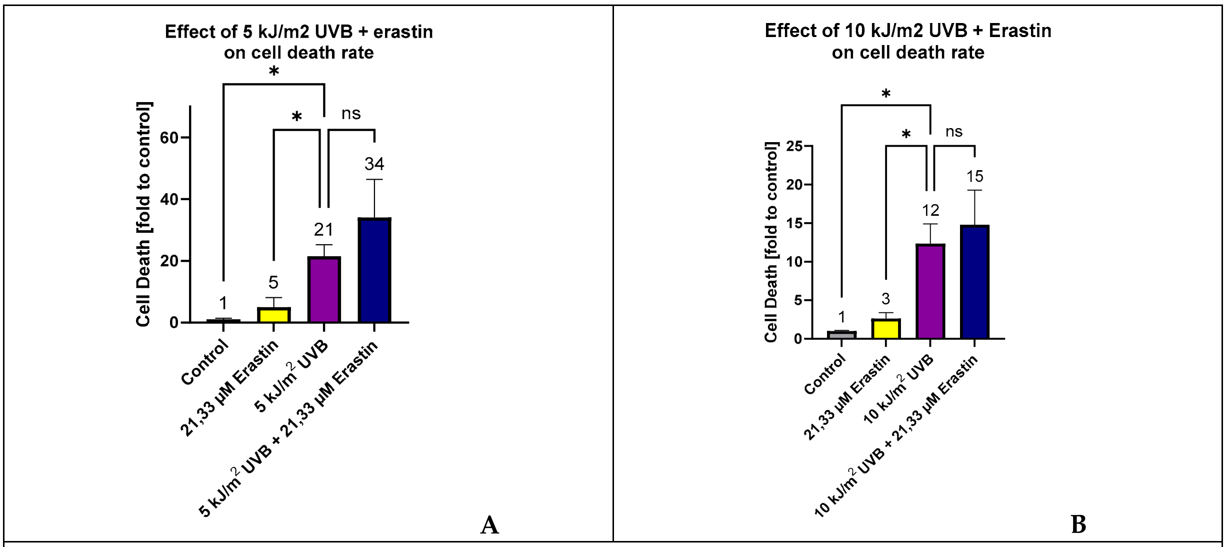


**Figure S3**. Combined effect of UVB irradiation and erastin on the rate of cell death. **A.** Effect of erastin at low (5 kJ/m²) intensities of UVB irradiation. **B.** Effect of erastin at high (10 kJ/m²) intensities of UVB irradiation. Cell death rate was determined by the release of LDH as described in method's section.

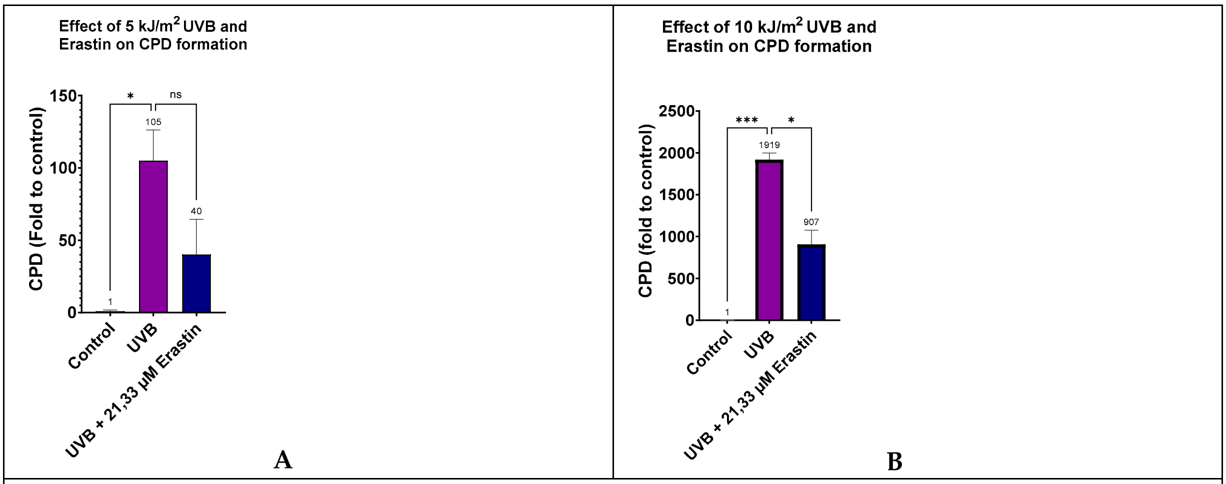


**Figure S4**. Effect of erastin on the elevated levels of CPD in keratinocytes exposed to 5 kJ/m² (**A**) and 10 kJ/m² (**B**) UVB irradiation. Inducers of ferroptosis decrease the levels of CPD by more than 2 fold, while the increase in the cell death rate upon treatment with erastin was not significant.
